# Friends in High Places? International Banks and the Argentinian Military Dictatorship

**DOI:** 10.1177/00220094251370755

**Published:** 2025-09-02

**Authors:** Carlo Edoardo Altamura

**Affiliations:** 5292 University of Manchester, UK; 27213 University of Lausanne, Switzerland

**Keywords:** commercial banks, international finance, dictatorship, internationalization

## Abstract

The presence of international banks in Argentina remained extremely stable throughout the 19th and 20th centuries. The situation changed dramatically after the military coup of 1976 as the number of international banks in the country rapidly increased from 15 in 1976 to 33 in 1982. The article will investigate the international financial relations of the last military junta by focusing on the relations of the regime with international commercial banks. The article will shed light on the role that private commercial banks played in the financing of the regime, in its ability to overcome international boycotts and, ultimately, to carry on its economic plan. First, the article will focus on the presence of international banks in Argentina since the second half of the nineteenth century. The second part will investigate the attitude of international banks towards Argentina in the 1960s and early 1970s to highlight how political and economic instability limited the efforts to establish solid financial relations with the country. Finally, the article will analyse the changing relationship between international banks and the Argentinian regime once the military ousted the Peronist government. We will focus on the perception of the new regime by international bankers, how new ventures in the country were planned and the attitude of the regime towards international finance to shed light on the role that non-state actors played in the consolidation of authoritarian rule.

The business of international banking in Argentina had remained relatively stable for more than a century. The first foreign bank to enter the country was the Bank of London and South America (initially known as the London, Buenos Ayres and River Plate Bank, then London and River Plate Bank in 1865) in late 1863. It was a commercial bank which financed imports and exports to and from Great Britain. Its stability was mainly due to the limited activity in land investments and mortgages. Although mainly geared toward the merchant community, the bank would also be active in government lending. Between 1912 and 1919, six other banks entered the country: *Sudameris* (formerly *Banco Francés e Italiano*), First National City Bank of New York, First National Bank of Boston, *Banco Holandés Unido*, *Banco Europeo América Latina* (formerly *Banco Italo-Belga*) and the Royal Bank of Canada. In the following years, marked by the Great Depression and World War II, the establishment of new banks declined substantially. The Italian bank Banco di Napoli established a branch in Buenos Aires in May 1930 to cater to the sizable Italian community in the country; in 1945 *Société Générale* from France acquired a majority stake in *Banco Supervielle* of Buenos Aires. In the 1950s, four banks established a presence in Argentina: Bank of Tokyo, *Banco do Brasil*, *Banco Alemán Transatlántico* (already present in Buenos Aires since 1887 but the branch was only re-opened in 1960), and Bank of America (Table 1).

In the 1960s, three more banks entered the country: *Banco de Santander* from Spain in 1963 and, between 1967 and 1969, the *Banco Central de España* and Chase Manhattan Bank. In the course of a century, thus, foreign presence in Argentina shows remarkable stability in terms of numbers of banks operating in the country, with a market dominated by a few banks from Europe and the US active in financing international trade and catering to the expats and immigrant communities. What happened after the military coup of 1976 is, consequently, somewhat extraordinary – and troubling – as the number of foreign banks operating in Argentina during the dictatorship increased dramatically, from 18 banks with 226 offices in 1976, to 32 banks with more than 340 offices in 1982 (Figure 1).

**Figure 1.**
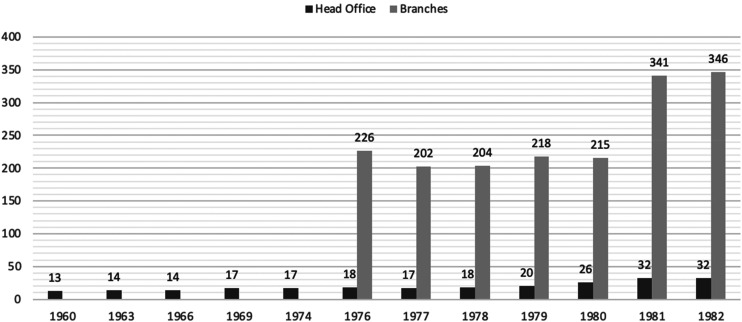
Foreign Banks in Argentina, 1960–82. Source: Ernesto Feldman and Juan Sommer, *Crisis financiera y endeudamiento externo en la Argentina*, Centro editor de América Latina, Buenos Aires, 1986, 108; Banco Central de la República Argentina, *Boletín Estadístico*, various years. NB: Data for branches not available before 1976.

According to Calcagno, foreign investments in the financial sector between March 1977 and December 1983 reached US$473 million, of which US$452 million were authorized during the period November 1978–November 1981.^1^ After a century of stable presence, the international banking scene in Argentina entered a period of frenzy with North American, French, British, Spanish but also Brazilian and Uruguayan banks entering the country. It is this striking banking expansion in a context of extreme political violence that this article will address.

This dramatic growth, as elaborated below, was both the result of international and domestic developments that the article will, at least partially, investigate. The present article is part of a growing body of academic literature addressing the role, or complicity, of non-state economic actors in supporting financially the Argentine junta.^2^

Horacio Verbitsky and Juan Pablo Bohoslavsky (p.1) insisted on the importance of including the role of ‘the individuals, bodies, and companies that supplied goods and/or services to the dictatorship or obtained benefits from it while they provided political support in return, thus consolidating the regime and facilitating the execution of its criminal plan.’

Unfortunately, data about commercial banks have been until recently extremely difficult to gather for obvious reasons, pushing the two scholars to argue that ‘there is no consolidated data on loan volume and lender identity.’^3^ In a similar vein, in a seminal work on the *Banco de la Nacion Argentina* (Argentina's state bank) and the Argentine dictatorship, Basualdo, Santarcangelo, Wainer, Russo and Perrone argue convincingly that the bank was the financial hand of the military regime but fail to address several crucial topics, for example how and why monetary and credit policies applied by the bank until the 1970s were overhauled by the Martinez de Hoz economic team or the interactions with the international financial community. Claudia Kedar and Raúl García Heras^4^ have made extensive use of archival material from multilateral organizations, such as the World Bank and the International Monetary Fund (IMF), to analyse their relationship with military regimes in the Southern Cone. García Heras has also devoted a book to the international financial relations of the Argentine regime but only limited attention is devoted to Western commercial banks and bankers.^5^ Despite the relevance of Heras and Kedar's work, it must be noted that most of the capital that Latin American authoritarian regimes, including Argentina, received during the 1970s did not come from official lenders but from private creditors, notably Western commercial banks. By 1985, commercial banks owned more than 50 percent of long-term debt in Latin America, compared with just 19 percent for bilateral and multilateral institutions.^6^ Victoria Basualdo et al. co-edited a seminal book on the Argentine dictatorship and big business insisting on the importance that international loans provided by global banks had in replacing productive investments with financial speculation but the role of international bankers is not thoroughly investigated.^7^

In the same volume, Juan Pablo Bohoslavsky addressed banking in Southern Cone dictatorships but no banking primary sources were used leaving the inner workings of the international banking world outside of his narrative.^8^

The availability of new archival material allows us to trace for the first time the international financial relations of the Argentine regime and the extent to which the international banking community sympathized with the military administration, particularly with its orthodox economic policies. The article will use primary sources from European commercial banks because, as pointed out by Altamura,^9^ European banking archives, unlike their US or Japanese counterparts, are accessible to external researchers allowing a more authoritative account of the business relations between military regimes and international financial actors. Besides, it must not be forgotten that European banks were the largest lenders to the Argentine public sector representing around 18 per cent of total loans compared to 16.9 per cent represented by US banks.^10^

At the international level, the increased presence of international banks in Argentina was the result of the gradual but steady internationalization of the geography of finance since the early 1960s and, especially, since the oil crisis of 1973.^11^

As Midland Bank reported in 1974:

The worldwide boom in commodity prices which characterised the year was overshadowed towards year-end by the enormous increases in the price of crude oil imposed by the producing countries. While the producers may find it difficult to maintain the price at its present level, there is bound, for the time being, to be a massive shift of purchasing power into their hands. The result, since they are unlikely to be able to spend more than a relatively small proportion on increased imports, will tend to be a dampening influence on world income and trade from the demand side, while at the same time pushing up costs still further in consuming countries…. In such circumstances a responsibility to ensure that the savings of the oil producers flow into productive use must fall on the national and international capital markets, and thereby offers an opportunity for the City of London.^12^

The internationalization of banking was already an existing process in the 1960s, especially for US banks trying to escape domestic regulations^13^ imposed in the early 1960s to counter the gradual balance of payment deterioration but several elements limited the extent of the process, including, to name a few, political and macroeconomic instability in the developing world, persistent capital controls and high information costs making international ventures a high risk investment.

In this sense, the oil crisis of 1973–74 did not create a new dynamic but it played a key role in accelerating, sometimes dramatically, existing tendencies. The oil crisis not only provided oil-producing countries with substantial amount of capital to invest but, at the same time, depressed economic demand in the West making overseas ventures much more palatable while the end of the Bretton Woods regime paved the way for a more financially liberal world. Quite suddenly, commercial banks became the central cog in the so-called recycling of petrodollars between surplus and deficit countries. International financial policymakers and domestic politicians, in fear of a potential global economic depression and, consequently, negative repercussions on domestic economies like in the 1930s, decided that oil surpluses could not and should not be fought.^14^ The Managing Director of the IMF, Johannes Witteveen, declared in May 1974: ‘Private markets have a basic role to play here, and it is to them that we must look for the main contribution in financing prospective balance of payments disequilibria.’^15^ A couple of years later, the Research Department of the IMF commented that: ‘commercial banks have been carrying out some of the functions which have often been thought of as more in the province of central banks, governments, and international organizations.’^16^

Argentina had already attracted the attention of Western bankers in search of profits and stability when the military took over during the so-called *Revolución Argentina* of 1966–73. In July 1970, two Barclays Bank officials were sent to Argentina to explore the possibility of establishing a presence in the country. In their country report, they explained: ‘The country was, for many years, held back by political instability, and a high rate of inflation, but President Ongania (lately deposed) created more stable conditions from 1966.’^17^ Under the new administration ‘most observers … are guardedly optimistic that stability will be maintained and that Unions will be kept sufficiently in check to prevent a renewal of rapid inflation (…)’. At the end of the visit, the officials recognized that ‘the country has great development potential…. The present regime is stable by Latin American standards and enjoys a good deal of popular support. It has men to tackle the economic problems that exist and there is determination not to slip back into the high inflationary era’. If this was not enough, there was ‘tolerance, no racial problems, and an air of liberty, even though there are no free elections and the military govern.’^18^

Although authoritarian politics offered certain attractions, the regime was weaker than it initially seemed and, starting from the late 1960s, Argentina entered a new period of political, social and economic crisis with inflation reaching 35 per cent in 1971 and protests in the streets of major industrial centres such as Cordoba.^19^ The instability in Argentina was also acknowledged by a leading French bank, *Crédit Lyonnais*, that reported that ‘the situation in Argentina does not allow us to expect any greater activity, despite our representative office in Buenos Aires.’^20^ The unstable situation under Allende in Chile, *Crédit Lyonnai*s concluded, also did not allow to expand their activities in the neighbouring country.

When a new – and much more brutal – military junta violently rose to power on 24 March 1976, it sought to make Argentina credible and creditworthy to the eyes of the international financial community. The regime appointed José Alfredo Martínez de Hoz as Minister of Economy, scion of a prominent family of cattle rancher, educated in English upper-class establishments (Eton College and Cambridge University), previously CEO of Argentina's largest steel company, Acindar. He had established links with the international financial community in the United States and United Kingdom.

A few months after his nomination, Martínez de Hoz appeared on the cover of the financial magazine Euromoney, in September 1976, as an indication of his prominence within international financial circles as a trustworthy actor who was able to make Argentina and the military regime a credible and creditworthy economic player. As reported by Euromoney:

Argentina is no longer a name to make international bankers shudder. Remembering Mao Tse-Tung's dictum that a journey of a thousand miles begins with the first step, the new Argentine regime had begun to put its economic house in order before its economics minister, Sr Martinez de Hoz, took off on his whirlwind tour of the world to drum up loans to meet Argentina's $1.2 billion debts that mature over the next few months. Although the minister's course was not entirely smooth, he did succeed in re-establishing an essential part of Argentina's image: credibility.^21^

As mentioned in the article, the main goal of the Argentine financial mission during its first visit to the financial community in the US and Europe was to put together a US$1.2 billion package to support the country's balance of payments. Ultimately, as the Euromoney magazine reported, Martínez de Hoz had been successful in putting together a US$400 million contribution from European sources, US$500 million from American banks and a US$300 million stand-by agreement with the IMF. David Rockefeller would later praise Martínez de Hoz as one of Argentina's ‘great ministers of the Economy’ with a ‘brilliant, solid, reasonable and realist’ programme.^22^

Despite the ongoing war against left-wing opposition movements and the persistent economic uncertainty during the first months of the dictatorship, the mood in the international financial community was markedly different. Martínez de Hoz toured the US and Europe making use of his respectability and European education to defend the regime's policies and deflect mounting concerns regarding human rights violations. When visiting Ted Rowlands in London, Minister of State for Foreign and Commonwealth Affairs, in July 1976, Martínez de Hoz, unconvincingly, responded to Rowlands when questioned about the political violence of the early months of the new regime that: ‘Problems arose for his Government when individuals disappeared and his Government genuinely did not know where they were (…).’^23^

Before meeting with Rowlands, Martínez de Hoz had been invited by the Chancellor of the Exchequer, Denis Healey, for a lunch with the financial community of London which included two influential bankers, Sir Jeremy Morse, Deputy Chairman of Lloyds Bank International (LBI), and Nicholas Baring of Baring Brothers.

Although ‘no matters of substance were discussed at the lunch’ the Chancellor told ‘Dr Martinez [de Hoz] that he had been watching with admiration what the Argentinians had been trying to do (…).’^24^ Baring and Morse also hosted a cocktail in honour of Martínez de Hoz at the prestigious Brooks Club on the final day of his visit to London. Apart from talks with ministers and the Governor of the Bank of England, this first visit of Martínez de Hoz to the UK also included meetings with British industrialists and, on July 20, meetings with the Chairman and directors of LBI, meetings with other bankers in the board room of LBI and meeting with representatives of Skandinaviska Enskilda Banken (SEB).

Lending to a violent and repressive military dictatorship was seen very early as potentially problematic. Guy Huntrods, LBI's director of the Latin American division noted: ‘The [Argentine] government must … walk a tightrope between the need for firmness and the danger of being branded by international opinion as repressive - a charge all too lightly banded around these days and very much à la mode in certain quarters only too ready to pass superficial and prejudiced judgements on Latin American countries where forms of government do not fit into the grey mould of social democracy and mediocrity which is their ideal.’^25^

In his report, Huntrods concluded in a way that summarizes well the attitude of the international financial community towards the junta during the early months of the regime:

In short, we are relieved by the change of government, pleased with the calibre of the economic team and with its general philosophy and objectives and encouraged by its performance so far…. Largely on our advice, it has been prepared to go to the IMF for help, and accept the required disciplines, and the presentation of its case to potential lenders has been well prepared and realistic. In consequence, a very substantial success has been achieved in USA and Europe. Our own role has been friendly, constructive and helpful.

He, then continued:

Somewhat characteristically the Argentines may have overestimated the enthusiasm and confidence abroad that the appearance of the new Government and especially its economic team has generated. If so, it is not entirely their fault, since as usual, and to some extent understandably, the foreign financial community and indeed the press in its sense of relief at the end of the Perón regime, overreacted. Now, three months later, much of the euphoria has evaporated and Argentina's prospects are seen as good, but fraught with difficulties, mostly of a structural socio-political nature, which will be extremely difficult to solve.^26^

The change of attitude is visible. The appreciation and the support for the junta's economic policies and the stability achieved at a high human cost clearly shifted the perceptions of international bankers and financiers. In its 1978–1980 plan for the Latin American region, *Crédit Lyonnais* put Argentina as one of its priorities, for the first time in years, together with Brazil, Mexico and Venezuela, explicitly because of the new military government that was open to foreign investments and was able to adjust public finances.^27^

The increased banking activity in authoritarian contexts in the developing world did not pass unnoticed, as Altamura and Kim have highlighted by putting the spotlight on two popular civic campaigns (Chile Solidarity Campaign and End Loans to Southern Africa) in Great Britain against banking activities in dictatorial Chile and racially-segregated South Africa.^28^ In June 1977, Barclays Bank International (BBI) Vice Chairman Steve Mogford, had been ‘asked by one our detractors [sic] whether we are “ploughing money into some of the military regimes in South America”’.^29^

To provide some guidance to such recurring grievances, BBI produced a report titled ‘Oppressive Regimes in Latin America’. The document reported that: ‘At present, democratic regimes are extremely rare in South America. If the definition of tyranny as the absence of the possibility to remove a government by peaceful means is accepted, practically all South American Governments are tyrannical (…).’ It is clear then, that the violent and tyrannical nature of South American regimes, including Argentina, was a well-known fact within the international financial community, especially since the United States cut off all military aid to the country in February 1977. The international press also reported extensively on human rights violations. The report continued by recognizing the regimes of the Southern Cone as the ‘most repressive’.

We might wonder whether lending to such a regime, once human rights violations and abuses were well-known and well-documented by the international media, could be considered as a legitimate business practice, an act of greed or as a deeply political act.

Bankers’ visits to the country intensified towards the end of the 1970s and the beginning of the 1980s. Sir Jeremy Morse, now Chairman of Lloyds Bank, visited Argentina in September 1977 for three days during a tour which included also visits to Brazil and Mexico. Morse reported that: ‘In the past eighteen months Argentina has staged one of its periodic sharp recoveries, after coming close to economic and social collapse under Peron's widow’.

He then continued:

The military government has mastered open terrorism at some cost [sic] in human rights; and their civilian Finance Minister, Martinez de Hos [sic], has freed the economy, brought inflation down from over 300% per annum (with a monthly peak near 1000%) to 150%, and created enough confidence to rebuild the reserves and get real growth up to 4–5%…. If one compares this recovery with the abortive one under Ongania and Krieger Vasena at the end of the ‘60s, the military backing for it looks stronger and more broadly based; and although it starts from a far worse position, Peronism is proportionately more discredited and Perón himself dead.

The prospects for Lloyds looked rosy under Martínez de Hoz's new economic scenario: ‘In our own business we seem to have taken good advantage of this first stage of recovery, in which positive real interest rates (at three-figure level!) and strong loan demand have enabled us to earn a real margin on funds of about 10 per cent. Pre-tax profits for 1976–77 will be near £10 million, of which £3 million should be remittable.’ Morse concluded his report by predicting further expansion in the country: ‘Given the government's policy of maintaining a positive real rate of interest – very different from the U.K. – there seems enough impetus in both the economy and the bank to justify the expansion.’^30^

The chief economist of *Société Générale*, Yves Laulan, was equally enthusiastic about Argentina's economic policies and decided to visit Argentina in person (and for the first time).

At the beginning of 1982, he was invited by Marcilio Marques Moreira of Unibanco, who later would become Brazilian Ambassador to the United States and Minister of Economy, Finance and Planning under President Collor, Laulan reported: ‘The problem of terrorism … appears to have been mastered. The methods were rigorous, even relentless, but they seem to have succeeded. Safety in Buenos Aires, seems to be remarkable day and night…. There is a climate of trust concerning the political situation…. Argentina continues to represent a good country risk for our bank.’^31^ The enthusiasm of *Société Générale* for the Argentine junta was reflected in the exceptional increase of total claims towards Argentina which increased by 446 per cent between 1978 and 1980.^32^ Enthusiasm for orthodox monetary policies and appreciation for strict control over dissent were reflected in persistent fears over the potential spread of Marxism.

In LBI's ‘Policy Guidance’ for 1980–81 it was clearly stated that: ‘In Latin America, the principal areas of disturbance will remain Central America and those islands in the Caribbean that are open to political influence from Cuba (…).’^33^

What proved especially attractive to the international financial community, apart from ideological considerations which are more complicated to prove, were the reforms that the economic team of the military junta tried to implement in the financial sector. Prior to 1977, the Argentine financial sector was heavily regulated; strict control over the financial sector had been reinforced in 1973, with the nationalization of deposits (partially amended in the following two years). Entry into the Argentine financial system was also heavily restricted as the Central Bank had to approve the establishment of new banks and the opening and closing of branches of existing banks. The military government radically reshaped the financial sector through two main laws: Law 21495, which authorized the Central Bank to convert the financial system back to a system of fractional reserve requirements, and Law 21526, which provided a new legal framework for financial institutions. These two laws provided the legal basis that allowed the Central Bank to de-regulate the Argentine financial sector, including interest rates which had been regulated since the mid-1930s.

Martínez de Hoz continued to tour the major capitals and the world financial centres to strengthen the links with political powers and the international financial community. After the visits of 1976, he travelled to Europe and the US multiple times.

During his tenure, he would eventually visit the UK four times, as an official guest or privately. In June 1980, the minister visited Western Germany to reciprocate the visit that Otto Graf Lambsdorff, Federal Minister for Economic Affairs, paid to Argentina in 1979. Martínez de Hoz was, according to the Head of the Eastern Latin American Department in the West German Ministry of Foreign Affairs ‘an interesting *Gesrprächspartner* [interlocutor]who was following sensible economic policies at home.’^34^

Importantly, despite the problems emanating from human rights violations, it was important for West German diplomats not to ‘drive Argentinians into a closer relationship with the Soviet Union or China by ostracising them over their human rights performance.’ Following his visit to West Germany, Martínez de Hoz visited France on 30–31 May 1980. The British Embassy in Paris reported that the Minister was received by President Giscard and had meetings with the ministers of Industry, External Trade, and Economy, as well as with executives of a number of French multinational companies. According to the Embassy: ‘The French press see Dr Martinez de Hoz's visit as part of the process of bringing Argentina in from the cold and regard it as inevitable that France with its known determination to boost exports, should be in the van of countries out to do business with Argentina.’^35^

An important point highlighted by this document is the importance of shoring up domestic industries and facilitate exports at a time when the world economy had been badly hit by two major oil crises in less than seven years. In this context, banks became crucial tools to extend credit facilities to developing countries, most of the time authoritarian regimes, so that they could buy manufactured goods from Western companies.^36^ After Bonn and Paris, the visit of Martínez de Hoz continued in the UK where he met the Chancellor on June 5. During his meeting, Martínez de Hoz argued that: ‘The two principal policies [of the military junta] had been to reduce state intervention in the economy and encourage the private sector.’ He went on and said that ‘there had been a general pruning of controls; price and foreign exchange controls had been abolished, whilst restraints on imports … had been relaxed. In the financial sector, interest rates, which, as a result of legal prescriptions, had previously been negative, had been allowed to reflect market forces.’^37^ In a letter to Michael Alexander, Thatcher's foreign affairs adviser, Martínez de Hoz was described by Roderic Lyne of the Foreign Office as ‘an international figure of considerable standing in the economic world’ whose thinking ‘is similar to ours’ and had made it clear that he would welcome ‘the participation of British industry in his plans for modernising and developing Argentina.’^38^ The Times reported in June 1980 that

No Argentine minister has achieved the international prestige of Dr José Alfredo Martínez de Hoz, who for the last four years has been responsible for the country's economy. In 1976 he took on his ministry in a bankrupt nation in the middle of a guerrilla war. By 1979 Argentina's reserves ranked eleventh in the world and bankers competed fiercely for the privilege of lending the country money.^39^

Nonetheless, the enthusiasm for the Argentine market was not without financial risks as increasing competition for this lucrative market pushed major commercial banks to neglect appropriate lending standards and due diligence. In June 1980, the Latin American department at LBI ‘were made aware that a number of serious bad debts were being incurred by various branches in Argentina.’ The bank's Chief Inspector was sent from London to Buenos Aires to investigate.^40^ The Chief Inspector reported that the ‘[Argentine] Circuit had come under some strain as a result of the expansion of business over the past few years. This expansion was achieved by an energetic and aggressive marketing policy which, in the competitive environment in Argentina, led to a relaxation of lending standards as branch managers struggled to produce the level of profits expected of them. The marketing thrust was reduced prior to the advent of the recession in late 1979, and managers were instructed to exercise greater caution in lending, but by then many future bad debts were already on the books.’ After this first investigation, LBI expected the extent of the bad loans to be known and the Chief Inspector was recalled to London in August 1980. Unfortunately for LBI, in September an increasing number of bad loans was still being reported, another team composed of the General Manager, Chief Inspector and senior inspectors was sent a second time to Buenos Aires in October 1980. The team reported that ‘the single most important factor leading to loss was the financial collapse of many sectors of commerce and industry brought about by the economic policies of the Government. However, in a number of cases bad and doubtful debt were attributable to errors of judgement and poor credit assessment…. The report drew attention to a number of control weaknesses in the credit assessment department and regional management (…).’ Revealingly though, Huntrods remarked in his report that: ‘I also believe that the aggressive marketing policy of the last five years has resulted in many but it has also produced, from October ‘75 to September ‘80, the considerable profit figure of £31mn, net of provisions and Head office adjustments (…).’

Following the liberalization policies of Martinez de Hoz, the macro-economic situation in Argentina by the early 1980s was becoming more complicated for international bankers as several banks went bankrupt, starting with the *Banco de Intercambio Regional* (BIR) in March 1980.^41^ BIR was historically a small regional bank which had expanded dramatically in a very short period of time, benefiting from the reforms of the regime. For example, its branch network had expanded from 46 branches in 1977 to 96 in 1979.

Just a few days after BIR's failure the Central Bank had to intervene three other major banks (*Banco Internacional*, *Banco Oddone*, *Banco de los Andes*), two of which were subsequently liquidated. The run was halted only by the intervention and commitment of Argentina's Central Bank to guarantee deposits up to 100 million Pesos, nonetheless, in the period 1980–82 more than 70 financial institutions had to be liquidated. Although a crisis was partially avoided, the Central Bank had to pay a high price to salvage the domestic financial system resulting in diminishing foreign currency reserves, rising inflation and public distrust.

Laulan of *Société Générale* reported after a trip to the US that: ‘I was impressed by the unanimous worries expressed by bankers concerning the debt situation in Latin America, especially with regard to Mexico, Argentina, Chile and also Brazil.’^42^ What was particularly worrisome, according to Laulan, was the fact that *Société Générale* was especially exposed to a limited number of Latin American countries and in the case of Argentina the large share of short-term loans. The situation for *Société Générale* was very similar to the one of many major global banks. Between 1978 and 1981, LBI's aggregated exposure to Brazil and Argentina grew from around £1 billion to around £1.6 billion, these two countries accounted for more than one quarter of LBI's total income. In the same period, Eurocurrency lending grew at an average growth rate of 37.7 per cent.

For *Société Générale* Latin American countries, essentially Mexico, Brazil, Panama, Venezuela and Argentina, accounted for 38 per cent of the total LDC (Socialist European countries excluded) exposure by year-end 1981 and by 1982 Argentina figured in eight place in terms of country-risk exposure with US$275 million. *Crédit Lyonnais* made an important acquisition in Argentina in 1981 by acquiring *Banco Tornquist* with 35 offices in the country (and 45 in Brazil) making of *Crédit Lyonnais* ‘the uncontested leading French bank in Latin America’.^43^

Argentina was an important but stagnant market for Western banks in the mid-twentieth century. As the article has illustrated, political and economic instability before 1976 did not allow new banks to enter the country on a substantial scale or, in the case of existing banks, to plan further investments or openings in the country. Under the new military junta, the number of foreign banks quickly doubled from 16 to 32. This expansion was accompanied by the accumulation of foreign debt which increased from US$7.8 billion in 1975 to US$45 billion in 1983, as illustrated in Table 2.^44^ What followed suit was the impossibility of Mexico to repay its foreign creditors in August 1982, the Debt Crisis, the ‘Lost Decade’ – which would affect most of Latin America and the Global South for the rest of the 1980s – and foreign banks more or less abruptly abandoning Latin America and the developing world under a pile of loss provisions (Table 2).^45^

**Table 1.** Foreign Banks in Argentina, 1863–1981.

Year	Bank	Country of origin
1863	Banco de Londres y América Latina del Sur	GB
1912–19	Banco Sudameris	France/Italy
	Citibank	USA
	The First National Bank of Boston	USA
	Banco Holandés Unido	The Netherlands
	Banco Europeo América Latina	Belgium/Italy
	The Royal Bank of Canada	Canada
1930	Banco di Napoli	Italy
1945	Supervielle Société Générale	France
1957–60	Bank of Tokyo	Japan
	Banco do Brasil	Brazil
	Deutsche Bank	West Germany
	Bank of America	USA
1963	Banco de Santander	Spain
1967–69	Banco Popular Argentino (Banco Central de España)	Spain
	Chase Bank	USA
1979–81	Banco Ambrosiano de América del Sud	Luxembourg
	Banque Nationale de Paris	France
	Barclays Bank International	UK
	Continental Illinois Bank	US
	Banco do Estado de Sao Paulo	Brazil
	Banco Exterior	Spain
	Banco Irving Autral	USA
	Banco Itau	Brazil
	Manufacturers Hanover Trust	USA
	Morgan Guaranty Trust	USA
	Banco Real de Sao Paulo	Brazil
	Republic National Bank of New York	USA
	Banco Tornquist (Crédit Lyonnais)	France
	Wells Fargo Bank	USA
	Banco Arfina	Argentina^a^
	Banco de la Repùblica Oriental de Uruguay	Uruguay

^a^Started operating in foreign currencies in December 1981.

**Table 2.** Pre-tax Profits Before Provisions and Loan Provisions of the UK Big Four, June 1987.

	NatWest	Barclays	Lloyds	Midland
Pre-tax profits before provisions (£m)	747	530	369	251
Growth on comparable period of previous year (%)	54	22	10	29
Provisions (£m)	496	570	1066	916

*Source:* Financial Times, ‘Barclays takes £40m loss after Third World loan provisions’, 31.07.1987.

**Table 3.** External Debt of Argentina, 1975–1985 (US$mn).

	Total	Public %	Private %
1975	7’875	51	49
1980	27’162	53	47
1982	43’634	66	34
1983	45’087	70	30
1984	46’903	77	23
1985	48’379	83	17

*Source:* Calcagno (1987).

Ultimately the defeat in the Malvinas (Falklands) War, the debt spiral and the economic crisis would bring down the junta putting an end to the brutal regime which until recently had benefited from the appreciation of the international financial community (Table 3).

The banking expansion has not been unnoticed but not thoroughly investigated by historians or social scientists. This is because the archives of commercial banks have not been used until recently to assess the role played by financial institutions during the military dictatorship.

This role cannot be underestimated as Argentina would not have been able to become the world's third most indebted country without the ability of the regime to borrow on international markets and the eagerness of Western bankers to lend.

How the money was spent would require further investigations and access to new sources but several authors have pointed out how the acquisition of military equipment played an important part in the country indebtedness. As reported by Altamura,^46^ arms imports to Argentina peaked in 1983 with more than US$1.3 billion following the signing of several agreements with Western countries in the second half of the 1970s. Not surprisingly, then, the *Estado Mayor General de la Armada* and the *Estado Mayor General del Ejército* all figured amongst the top debtors with respectively US$2 billion and US$700 million.^47^

Why banks established increasingly closer links with a brutal military dictatorship is beyond the scope of a single article as the answer lies in both domestic and international elements.

Looking at the archival evidence currently available, it is difficult not to see a sense of relief when the military took power, as political instability and labour unrest were seen as major factors behind Western banks’ reticence to scale up their involvement in the country. Nonetheless, the single most important domestic factor behind the return of Argentina on the international financial scene was the prestige of Martinez de Hoz, his connections in the United States and Europe, especially the United Kingdom where he had been educated and the reassurances that he offered to international bankers. If Argentina found in Martínez de Hoz a respectable financial ambassador and an efficient ‘information broker’^48^ willing to ignore human rights violations to market Argentina as a creditworthy client, Western bankers also were eager lenders after the two oil crises, which had flushed them with gargantuan amounts of money, the end of the Bretton Woods regime and the expansion of the un-regulated Euromarket, especially the medium-term syndicated Euroloans.^49^

In the depressed economic context of the 1970s, commercial banks became crucial vehicles to help domestic industries finding new clients by extending attractive financing packages to developing countries.^50^ Competition between governments became fierce and, consequently, competition between commercial banks intensified. Although archival sources currently available allow us to identify individual preferences for political stability, fiscally and monetary conservative regimes and rejection of Marxism, commercial bankers did not seem to be, overall, influenced by ideology in their lending decisions. International competition, greed and political pressures to facilitate the recycling of petrodollars proved to be sufficient elements to push commercial banks into the arms of a brutal regime. Whether this absolves bankers from the (in)direct complicity in the crimes committed by the military junta is a matter for discussion.

## Notes

1 Eric Calcagno, ‘Los banco transnacionales y el endeudamiento externo en la Argentina’, *Cuadernos de la CEPAL*, 21 November 1987.

2 Juan Pablo and Opgenhaffen Veerle, ‘The Past and Present of Bank Responsibility for Financing the Argentinean Dictatorship’, *Harvard Human Rights Journal*, 23 (2010), 157–203; Horacio Verbitsky and Juan Pablo Bohoslavsky (eds), *The Economic Accomplices to the Argentine Dictatorship. Outstanding Debts* (Cambridge and New York 2015); Eduardo Basualdo, Juan Santarcangelo, Andrés Wainer, Cintia Russo and Guido Perrone (eds), *El Banco de la Nacion Argentina y la Dictadura. El Impacto de la Transformaciones Economicas y Financieras en la Politica Crediticia (1976–1983)* (Buenos Aires 2016).

3 Juan Pablo Bohoslavsky, ‘The Complicity of the Lenders’, in Horacio Verbitsky and Juan Pablo Bohoslavsky (eds), *The Economic Accomplices to the Argentine Dictatorship. Outstanding Debts* (Cambridge and New York 2015), 107.

4 For example, Claudia Kedar, ‘Human Rights and Multilateral Lending; The World Bank, Argentina and the United States, 1976–1978’, *International History Review*, 41, 6 (2019), 1256–75; Kedar, ‘IMF and the Chilean Chicago Boys’; Kedar, ‘Economic Neutrality during the Cold War: The World Bank, the United States, and Pinochet’s Chile, 1973–1977’, *Cold War History*, 18, 2 (2018), 149–67; Raúl García Heras, ‘Multilateral Loans, Banking Finance, and the Martinez de Hoz Plan in Argentina, 1976–1981’, *Revista de Historia Económica*, 36, 2 (2018), 215–40; and García Heras, ‘The Return of International Finance and the Martínez de Hoz Plan in Argentina, 1976–1978’, *Latin American Research Review*, 53, 4 (2018), 799–814.

5 Raúl García Heras, *Deuda Externa, Crisis y Política. Banqueros, Fondo Monetario y Banco Mundial en la Argentina 1973–1983* (Buenos Aires 2022).

6 Reported in Carlo Edoardo Altamura, ‘Global Banks and Latin American Dictators, 1974-1982’, *Business History Review,* 95, 2 (2021), 301–32.

7 Victoria Basualdo, ‘Business and the Military in the Argentine Dictatorship (1976-1983): Institutional, Economic, and Repressive Relations’, in V. Basualdo, H. Berghoff and M. Bucheli (eds), *Big Business and Dictatorship in Latin America* (Cham, Switzerland 2021).

8 Juan Pablo Bohoslavsky, ‘Banking Southern Cone Dictatorships’, in V. Basualdo, H. Berghoff and M. Bucheli (eds), *Big Business and Dictatorship in Latin America* (Cham, Switzerland 2021).

9 Idem., 306.

10 Eric Calcagno, ‘Los bancos transnacionales y el endeudamiento externo en la Argentina’, *Cuadernos de la CEPAL* (1987).

11 For example, Carlo Edoardo Altamura, *European Banks and the Rise of International Finance: The Post-Bretton Woods Era* (London 2016).

12 Midland Bank Archives, MBA, Annual Report 1974.

13 These measures included the Interest Equalization Tax (1963), the Voluntary Foreign Credit Restraint Program (1965) and the Foreign Direct Investment Program (1968).

14 On the recycling see, for example, Altamura (2016) and Altamura and Flores Zendejas (2021).

15 International Monetary Fund Archives (IMFA), EURAI Subject Files Box 145, Speech of Johannes Witteveen at the Economic Club of Detroit, 4 May 1974.

16 IMFA, WHDAI Economic Subject Files Box 25, Discussion Paper «International Commercial Banking and World Economic Stability », 14 September 1976.

17 Barclays Bank Archives (BBA), BBA, 80/3173, Report on Barclays Group Representation in Argentina, 13–22 July 1970.

18 BBA, 80/3173, ‘Visit to South America by Mr G.G. Money and D. V. Weyer’ 13 July 1970–5 August 1970.

19 J. C. Portantiero, ‘Economía y política en la crisis Argentina: 1958-1973’, *Revista Mexicana de Sociología*, 39, 2 (1977), 558.

20 Crédit Lyonnais Archives (CLA), 110AH10, Direction Centrale des Affaires Internationales, Programme 1973-75, 14 November 1972.

21 *Euromoney*, ‘Argentina Takes the First Step on the Long March to Recovery’, September 1976.

22 *Gente*, ‘Lo que pienso de Martínez de Hoz’, 6 April 1978.

23 The National Archives (TNA), FCO7/3031, Record of Meeting between the Minister of State for Foreign and Commonwealth Affairs and the Argentine Minister of Economy, Held at the FCO at 3:30PM on Monday 19 July, 1976.

24 TNA, FCO7/3031, Lunch with the Argentine Minister of Economy, 19 July 1976.

25 Lloyds Bank Archives (LBA), HO/Ch/Fau/44, ‘The Visit to London of the Argentine Financial Mission’, July 1976.

26 Idem.

27 SGA, 110AH11, Programme 1978–80: Zone Amérique Latine/Grèce/Portugal, 6 October 1977.

28 Carlo Edoardo Altamura and Seung Woo Kim, ‘Exclusionary Regimes, Financial Corporations, and Human Rights Activism in the UK, 1973–92’, *The International History Review*, 45, 5 (2023), 787–806.

29 BBA, 80/6284, Note Oppressive Regimes in South America, 23 June 1977.

30 LBA, HO/Ch/Mor/63, Note of Sir Jeremy Morse on His Visit to Argentina, 11 October 1977.

31 SGA, 8118, Voyage Argentine et Brésil, 31 March 1980.

32 SGA, 81093, Note sur l’activité internationale de la Société Générale, 31 July 1981.

33 LBA, F/Ce/Off/2.1, LBI Policy Guidance 1980-81, April 1980.

34 TNA, FCO7/3727, Argentinian Minister of the Economy, 6 June 1980.

35 TNA, FCO7/3727, Visit to France of Argentinian Minister of Economy, 9 June 1980.

36 On this topic see: Carlo Edoardo Altamura and Juan Flores Zendejas, ‘Politics, International Banking, and the Debt Crisis of 1982’, *Business History Review*, 94, 4 (2021).

37 TNA, FCO7/3727, Note of a Meeting Held at No.11, Downing Street at 6:15PM on Thursday, 5^th^ June 1980.

38 TNA, FCO7/3727, Letter from R. M. J Lyne to Michael Alexander, 26 February 1980.

39 The Times, ‘Argentina’s Economic Hard Man Upsets the Military’, 3 June 1980. On De Hoz’s policies see: Raúl García Heras, ‘The Return of International Finance and the Martínez de Hoz Plan in Argentina, 1976-1978’, *Latin American Research Review*, 53, 4 (2018), 799–814.

40 LBA, F1/D/Boa/3.1, Argentina: Bad and Doubtful Debts, 15 January 1981.

41 On the crisis of 1980 see for example: T. J. T. Baliño, ‘The Argentine Banking Crisis of 1980’, in *Banking Crises* (Washington D.C. 1991).

42 SGA, 81145, Note à l’attention du Président, 15 February 1982.

43 *Le Monde*, ‘Crédit Lyonnais : un formidable rattrapage’, 28 May 1982.

44 Calcagno, 1987, 39.

45 The literature on the Debt Crisis is vast. See for example: William R. Cline, *International Debt: Systemic Risk and Policy Response*, Institute for International Economics (Washington D.C. 1984); William John Darity and Bobbie L. Horn, *The Loan Pushers. The Role of Commercial Banks in the International Debt Crisis* (Cambridge, MA 1988); Robert Devlin, *Debt and Crisis in Latin America : the Supply Side of the Story* (Princeton 1989); Jeffry Frieden, ‘Third World Indebted Industrialization: International Finance and State Capitalism in Mexico, Brazil, Algeria, and South Korea’, *International Organization*, 35, 3 (Summer 1981), 407–31; David F. Lomax, *The Developing Country Debt Crisis* (London 1986); Michael Moffitt, *The World’s Money: International Banking from Bretton Woods to the Brink of Insolvency* (New York 1983); Jeffrey Sachs (ed.), *Developing Country Debt and the World Economy* (Chicago, IL 1991); Jerôme Sgard, *The Debt Crisis of the 1980s: Law and Political Economy* (London 2023).

46 Carlo Edoardo Altamura, ‘Global Banks and Latin American Dictators, 1974-1982’, *Business History Review*, 95, 2 (2021), 327–8.

47 Calcagno (1987), 63.

48 Paula Vedoveli, 'Information Brokers and the Making of the Baring Crisis, 1857–1890', *Financial History Review,* 25 (2018), 357–86.

49 On the Euromarket see, for example: Catherine R. Schenk, ‘The Origins of the Eurodollar Market in London: 1955–1963’, *Explorations in Economic History*, 35, 2 (1998), 221–38; Stefano Battilossi, Youssef Cassis, and Kazuhiko Yago (eds), *Handbook of the History of Money and Currency* (Singapore 2019); Schenk, *The Decline of Sterling* (Cambridge, UK 2010); Gary Burn, *The Re-Emergence of Global Finance* (Basingstoke 2006); Carlo Edoardo Altamura, *European Banks and the Rise of International Finance: The Post-Bretton Woods Era* (London 2016); Altamura, ‘The Paradox of the 1970s: The Renaissance of International Banking and the Rise of Public Debt’, *Journal of Modern European History*, 15, 4 (2017), 529–53.

50 Carlo Edoardo Altamura and Juan Flores Zendejas, ‘Politics, International Banking, and the Debt Crisis of 1982’, *Business History Review*, 94, 4 (2021).

